# Sod1 Deficiency Reduces Incubation Time in Mouse Models of Prion Disease

**DOI:** 10.1371/journal.pone.0054454

**Published:** 2013-01-22

**Authors:** Shaheen Akhtar, Julia Grizenkova, Adam Wenborn, Holger Hummerich, Mar Fernandez de Marco, Sebastian Brandner, John Collinge, Sarah E. Lloyd

**Affiliations:** Medical Research Council Prion Unit and Department of Neurodegenerative Disease, University College London Institute of Neurology, London, United Kingdom; Ohio State University, United States of America

## Abstract

Prion infections, causing neurodegenerative conditions such as Creutzfeldt-Jakob disease and kuru in humans, scrapie in sheep and BSE in cattle are characterised by prolonged and variable incubation periods that are faithfully reproduced in mouse models. Incubation time is partly determined by genetic factors including polymorphisms in the prion protein gene. Quantitative trait loci studies in mice and human genome-wide association studies have confirmed that multiple genes are involved. Candidate gene approaches have also been used and identified *App*, *Il1-r1* and *Sod1* as affecting incubation times. In this study we looked for an association between *App*, *Il1-r1* and *Sod1* representative SNPs and prion disease incubation time in the Northport heterogeneous stock of mice inoculated with the Chandler/RML prion strain. No association was seen with *App*, however, significant associations were seen with *Il1-r1* (P = 0.02) and *Sod1* (P<0.0001) suggesting that polymorphisms at these loci contribute to the natural variation observed in incubation time. Furthermore, following challenge with Chandler/RML, ME7 and MRC2 prion strains, Sod1 deficient mice showed highly significant reductions in incubation time of 20, 13 and 24%, respectively. No differences were detected in Sod1 expression or activity. Our data confirm the protective role of endogenous Sod1 in prion disease.

## Introduction

Prion diseases or transmissible spongiform encephalopathies (TSEs) are progressive neurodegenerative diseases which are invariably fatal. They include Creutzfeldt-Jakob disease (CJD) in humans, bovine spongiform encephalopathy in cattle (BSE) and scrapie in sheep and goats [1]. They are transmissible misfolded protein diseases caused by the conversion of normal cellular prion protein (PrP^C^) to abnormal isoforms, known as PrP^Sc^. In addition to PrP^Sc^ accumulation they are characterised by spongiform vacuolation, gliosis and neuronal loss in the brain.

Prion disease incubation time in experimental mouse models is remarkably consistent if experimental parameters are kept constant, however, there is considerable variation between different inbred strains of mice suggesting a strong genetic contribution. The prion protein gene, *Prnp*, is the major component where homozygous knockout mice are completely resistant to prion propagation and disease, hemizygous knockout mice have a prolonged incubation time and PrP overexpressors have a much shorter incubation time than wild type mice [2], [3]. Incubation time is also modulated by a naturally occurring polymorphisms in *Prnp* where *Prnp^a^* (108-Leu, 189-Thr) and *Prnp^b^* (108-Phe, 189-Val) are associated short and long incubation times respectively [4]–[8]. Similarly, a methionine to valine polymorphism at codon 129 of human PrP is also a major susceptibility factor for human prion disease [9]–[13]. Most inbred lines of mice are *Prnp^a^* and within these there is still considerable variation in incubation time thus implicating other genes [14]. Quantitative trait loci study in mice [15]–[19] and genome-wide association studies (GWAS) in humans [13], [20] suggest that although *Prnp* is the single most important factor, the combined effect of several other genes are also of importance.

In addition to traditional mapping techniques, individual candidate gene approaches have also been employed to identify genes that influence prion disease incubation time. Twenty candidate genes were screened based on pathways and genes previously implicated in prion disease by testing knockout or transgenic mouse models [21]. Under these conditions, most genes had no effect on incubation time, however, knockout of *App* (amyloid precursor protein), *Il1-r1* (interleukin 1 receptor 1) and overexpression of human *SOD1* (superoxide dismutase 1) increased survival by 13, 16 and 19% respectively.

To test whether *App*, *Il1-r1* and *Sod1* contribute to the naturally occurring variation in inbred lines of mice we used a heterogenous stock (HS) of mice inoculated with the Chandler/Rocky Mountain Laboratory (RML) mouse-adapted scrapie prion strain to look for a statistical association between prion disease incubation time and these genetic loci [22]–[25]. The *Sod1* locus produced a highly significant association therefore we investigated this further by challenging *Sod1* deficient mice (*Sod1^−/−^*) with three different prion strains.

## Materials and Methods

### Ethics Statement

All procedures were conducted in accordance with institutional, UK and international regulations and standards on animal welfare and conform to ARRIVE guidelines [26]. Ethical approval was granted by the Medical Research Council (MRC) Prion Unit ethics committee and carried out under UK Home Office licence PPL70/7274.

### Mice

Northport HS mice (Gift from R. Hitzemann, Portland, Oregon, USA) were bred and prion incubation times were collected for mice at generation 37 (n = 1052) as previously described [22]. *Sod1* knockout mice (B6;129S7-Sod1^tm1Leb^/J) were obtained from the Jackson Laboratory (Bar Harbor, Maine, USA) and backcrossed to C57BL/6J to >N7 (Gift from EMC Fisher, University College London Institute of Neurology, London, UK) [27]. The resulting heterozygote animals (*Sod1^−/+^*) were intercrossed to provide homozygote knockouts (*Sod1^−/−^*) and wild type litter mate controls (*Sod1^+/+^*). Other inbred mouse lines were provided by Harlan, UK Ltd. (Bicester, UK).

### Prion Inoculation and Phenotyping

Inocula were made from the brains of terminally sick mice as 1% (weight/volume) homogenates in D-PBS. The Chandler/RML (I9900), ME7 (I9459) and MRC2 (I9468) prion strains were sourced and prepared as previously described [22], [28]. Mice were anaesthetized with isofluorane/O_2_ and inoculated intra-cerebrally into the right parietal lobe with 30 µl of inocula as previously described [15]. To minimise animal suffering, mice were examined daily for clinical signs of prion disease and were culled once a definitive diagnosis had been made or earlier if showing any signs of distress or other health problems. Mice were killed by exposure to carbon dioxide gas in rising concentration. Criteria for defining scrapie in mice were as previously described [29]. Incubation time was defined as the number of days from inoculation to diagnosis. All procedures were conducted in accordance with institutional, UK and international regulations and standards on animal welfare and conform to ARRIVE guidelines [26]. Ethical approval was granted by the MRC Prion Unit ethics committee and carried out under UK Home Office licence PPL70/7274.

### PCR and Sequencing

DNA for each of the heterogeneous stock parental lines was obtained from the Jackson Laboratory (Bar Harbor, Maine, USA). PCR and sequencing reactions were carried out as previously described [23] and run on a 3730 capillary sequencer (Applied Biosystems).

### Genotyping

DNA was extracted from the HS cross mice as previously described [23]. SNP genotyping was carried out using the Allelic Discrimination function on a 7500 Fast Real-time PCR machine (Applied Biosystems) using RoxMegaMix Gold (Microzone, Ltd) as previously described [23]. Primers and probes are shown in Table S1.

### Real-time RT-PCR

Whole brain RNA was extracted from uninfected (6–8 weeks old) mice and reverse transcribed using AMV reverse transcriptase and random primers as previously described [29]. Control reactions were carried out with no reverse-transcription for each sample to ensure no genomic DNA contamination of the RNA preparation. Real-time RT-PCR reactions were carried out on a 7500 Fast Real-time PCR System (Applied Biosystems) as previously described [29]. *Sod1* Taqman Gene Expression assay (Life Technologies) was duplexed with each of three endogenous controls (*GAPDH*, *β-actin* and *Thy-1)* [23]. All reactions were carried out in triplicate. All data passed a normality test and was statistically evaluated using a t-test.

### Western Blotting

For detection of PrP^Sc^ by western blotting 10% (weight/volume) brain homogenates in D-PBS were prepared by ribolysing. Samples were benzonase treated and proteinase K digested (50 µg/ml of proteinase K for 1 h at 37°C) and blotted as described previously [30]. PrP was detected with anti-PrP monoclonal antibody ICSM35 (D-Gen Ltd, UK) [31] and alkaline-phosphatase-conjugated anti-mouse IgG secondary antibody (Sigma-Aldrich) developed in the chemiluminescent substrate CDP-Star (Tropix Inc). For detection of Sod1, samples were prepared as above but without proteinase K digestion and detected with a rabbit polyclonal antibody (0.5 ug/ml) (Abcam). Anti-β-actin mouse monoclonal antibody (0.05 ug/ml) (Sigma) was used as an internal control as previously described [28].

### Immunohistochemistry

Mouse brains were fixed in 10% buffered formal saline (BFS) and prion infected tissue was treated in 98% formic acid for one hour to remove infectivity. Tissues were paraffin wax embedded, sectioned and stained as previously described [29]. Sections were stained with haematoxylin and eosin (H&E) for general viewing including assessment of spongiosis and neuronal loss. Disease associated prion deposition was visualised with anti-PrP monoclonal antibody ICSM35 (D-Gen Ltd, UK) and gliosis was determined with an anti-glial fibrillary acid protein (GFAP) antibody (Dako Ltd, UK).

### Quantification of PrP^c^

10% (weight/volume) brain homogenates were used to measure endogenous levels of PrP^c^ by enzyme linked immunosorbent assay (ELISA) as previously described [32]. Total protein concentration was measured using a Pierce BCA protein assay kit (Thermo Scientific) according to the manufacturer’s instructions.

### Superoxide Dismutase Enzymatic Activity Assay

Total SOD activity was measured in 10% weight/volume (20 mM HEPES, pH 7.2, 1 mM EGTA, 210 mM mannitol, 70 mM sucrose) brain homogenates using a SOD assay kit (Calbiochem) according to the Manufacturer’s instructions. A Coomassie (Bradford) protein assay kit (Bio-Rad) was used to measure the total protein concentration according to the manufacturer’s instructions.

### Statistical Analysis

Statistical tests were carried out using GraphPad InStat (GraphPad Software, Inc, California, USA) and SPSS (IBM). The Kaplan-Meier log-rank test was used to analyse survival data.

## Results

### Association Studies

To look for an association between *App*, *Il1-r1*, *Sod1* and prion disease incubation time we used the Northport HS of mice as previously described [22], [23], [25], [33]. The stock was generated from eight parental lines (A/J, AKR/J, BALB/cJ, C3H/HeJ, C57BL/6J, CBA/J, DBA/2J and LP/J) and n = 1052 mice from generation 37 were inoculated with the Chandler/RML mouse adapted scrapie prion strain [22].

Inbred lines of mice are closely related, however, it is expected that multiple single nucleotide polymorphism (SNPs) will be present between the parental lines of the HS mice. To select the most advantageous SNPs for genotyping we sequenced the coding region, untranslated regions (UTR), putative promoters (as predicted by PROSCAN (www-bimas.cit.nih.gov/molbio/proscan) and flanking intronic sequence. From this data we defined the major strain distribution pattern (SDP) across the parental lines. This was subsequently confirmed by comparison with genomic sequencing data SNPs generated by the Sanger Institute (www.sanger.ac.uk/cgi-bin/modelorgs/mousegenomes/snps.pl) which covered the entire gene starting from 5 kb upstream of the 5′UTR. The main SDP for each locus and the total number of SNPs is shown in table 1. To capture each SDP, one tagging SNP was chosen for genotyping (Table 2). For one SDP of *App* and *Il1-r1,* SNPs were chosen based on putative functional significance (GCF binding site and amino acid change respectively). Other SNPs were chosen based on their ability to tag the SDP. Genotyping was carried out in approximately 400 mice representing the extremes of the incubation time distribution as this captures most of the power of the cross [25]. For details of primers and probes see Table S1. For *App*, two SNPs were tested, representing two SDPs (Tables 1 and 2). rs47601325 is located in the promoter within a GCF binding site (PROSCAN) and rs46934600 is within intron 2 and is not known to be a functional variant. Neither of these SNPs showed an association with incubation time (Table 2). One non-synonymous *Il1-r1* coding polymorphism (Ala71Val) from exon 3 (Table 2) was tested and this showed a significant association with incubation period (P = 0.02, Kruskal-Wallis non-parametric ANOVA) where the CC genotype was associated with a short incubation time (144±1.6, mean (days) ± sem) and the TT genotype with a longer incubation time (155±3.3). The heterozygous allele CT was not significantly different from CC (145±1.7) suggesting a dominant mode of action. An allelic test (Mann-Whitney) also showed a significant association (P = 0.007, C allele 144±1.0 and T allele 149±1.4) (Table 2). A *Sod1* SNP from intron 3 (Table 2), of no known function, also showed a highly significant association with prion disease incubation time (P<0.0001, Kruskal-Wallis non-parametric ANOVA) where the GG genotype was associated with a short incubation time (132+4.7) and the AA genotype with a longer incubation time (149+1.5). In contrast to *Il1-r1*, the *Sod1* alleles appear to behave in an additive way with the heterozygous allele GA having an intermediate mean incubation time (141±1.8). The allelic test (Mann-Whitney) also showed a highly significant association (P<0.0001, G allele 140±1.7 and A allele 147±1.0) (Table 2).

**Table 1:** Major strain distribution patterns genotyped for HS mice.

Genes	Allele and strain distribution pattern	SNPs
*Sod*	A = (A, BALB, C3H, C57, LP); G = (AKR, CBA, DBA)	15/15
*App*	C = (A, C3H, DBA); G = (AKR, BALB, C57, CBA, LP)	413/978
	C = (A, BALB, C3H, CBA, DBA); T = (AKR, C57, LP)	303/978
*Il1-r1*	C = (A, AKR, BALB, C57, C3H, CBA, DBA); T = (LP)	70/81

The number of SNPs is taken from genomic sequence generated by the Sanger Institute (www.sanger.ac.uk/cgi-bin/modelorgs/mousegenomes/snps.pl) and spans 5 kb upstream of the 5′UTR start site to the end of the 3′UTR (NCBI Build 37). Ambiguous SNPs have been excluded. The strain distribution pattern (BALB, CBA); (A, AKR, C3H, C57, DBA, LP) was also seen for *App* (239/978) but this was not genotyped in the HS mice. Other individual strain distribution patterns were seen ≤5 times.

**Table 2:** SNP genotyping in HS mice.

Gene	SNP	Genotypic test	Allelic test
		p-value (n)	p-value
*Sod1*	Inron 3 A/G Chr16 90,224,725	<0.0001 (386)	<0.0001
*App*	Promoter C/G *rs47601325* Chr16 85,174,122	0.12 (406)	0.85
*App*	Intron 2 C/T *rs46934600* Chr16 85,103,728	0.92 (411)	0.92
*Il1-1r*	Exon 3 Ala71Val C/T Chr1 40,350,109	0.02 (416)	0.01

Genomic location is given based on mouse genome assembly NCBI build 37. Details of the *Sod1* and *Il1-r1* SNPs are available from the Sanger Centre (www.sanger.ac.uk/cgi-bin/modelorgs/mousegenomes/snps.pl). All polymorphisms were analysed by allele discrimination using a 7500 Fast real time PCR system (Applied Biosystems). For probe details see Table S1. For all genotypes, the statistical test used was the Kruskal-Wallis non-parametric ANOVA. The allelic test used was the Mann-Whitney test.

### *Sod1* mRNA Expression

**Figure 1:**
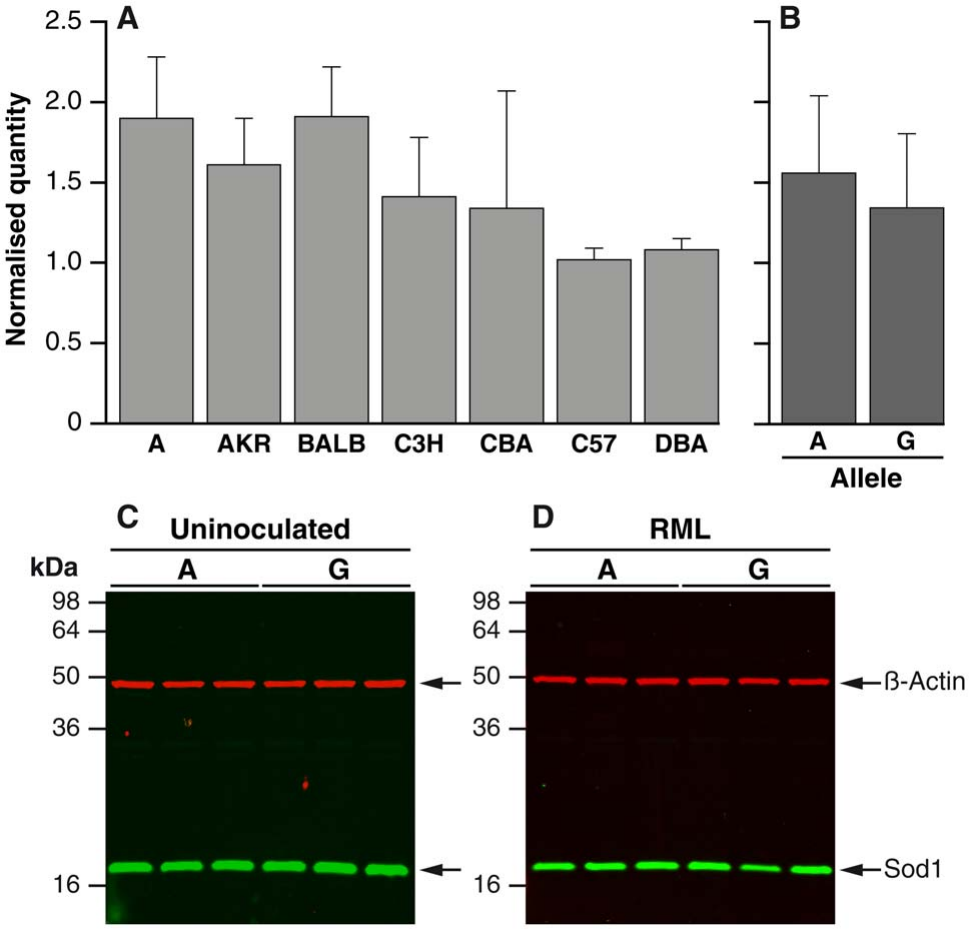
*Sod1* expression in mouse brain. Panel A and B. Quantification of *Sod1* mRNA expression in whole brain by real-time RT-PCR. N = 6 for all groups and samples were run in triplicate. All samples were duplexed for *Sod1* (Fam-label) and an endogenous control *GAPDH, β-actin* or *Thy-1* (Vic-label). Expression level is expressed in arbitrary units as normalised by the geometric mean of the quantity of the endogenous controls (*y*-axis). Error bars represent the standard deviation. (A) *Sod1* mRNA expression level for parental strain of the HS mice (except LP). (B) *Sod1* mRNA expression level grouped by allele (A/G) (A = A, BALB, C3H, C57; G = AKR, CBA, DBA). No significant difference was observed between the groups. Panels (C) and (D). Quantification of Sod1 protein in whole brain (10% homogenate, weight/volume) from n = 3 A allele mice (C57Bl/6) and G allele mice (FVB/N). FVB/N mice were used to represent a G allele strain rather than an HS parental strain due to availability of tissue. Samples were immunoblotted with rabbit polyclonal anti-human SOD1(Abcam) and detected with IRDye800CW (green) conjugated goat anti-rabbit IgG (Li-Cor). Anti-β-actin mouse monoclonal antibody (Sigma) was also included as a loading control and was detected using IRDye680 (red) conjugated goat anti-mouse IgG (Li-Cor). Fluorescence was visualised using an Odyssey infra-red imager (Li-Cor). (C) Uninoculated mice, (D) Terminally sick Chandler/RML prion inoculated mice.

Based on the association study data *Il1-r1* and *Sod1* loci are associated with prion disease incubation time, however, *Sod1* has the strongest effect with a mean difference of 17 days between the homozygous genotypes compared to 11 days for the *Il1-r1* genotypes, therefore, we only followed up the *Sod1* data. No known functional polymorphisms were found in the HS parental strains for *Sod1* and so we measured *Sod1* mRNA expression levels in uninfected brains from the parental lines of the HS (except LP) to see whether endogenous expression level was associated with genotype. *Sod1* mRNA expression levels were quantified using real time RT-PCR. Although variation between the inbred lines was seen (Figure 1A), when grouped by *Sod1* genotype (A = A, BALB, C3H, C57; G = AKR, CBA, DBA) this was not significant (Figure 1B). We also saw no difference in Sod1 protein expression by western blot between A and G allele mice either in uninfected or Chandler/RML infected mice (Figure 1C–D).

### Transmission of Prions to *Sod1^−/−^* Mice

**Figure 2:**
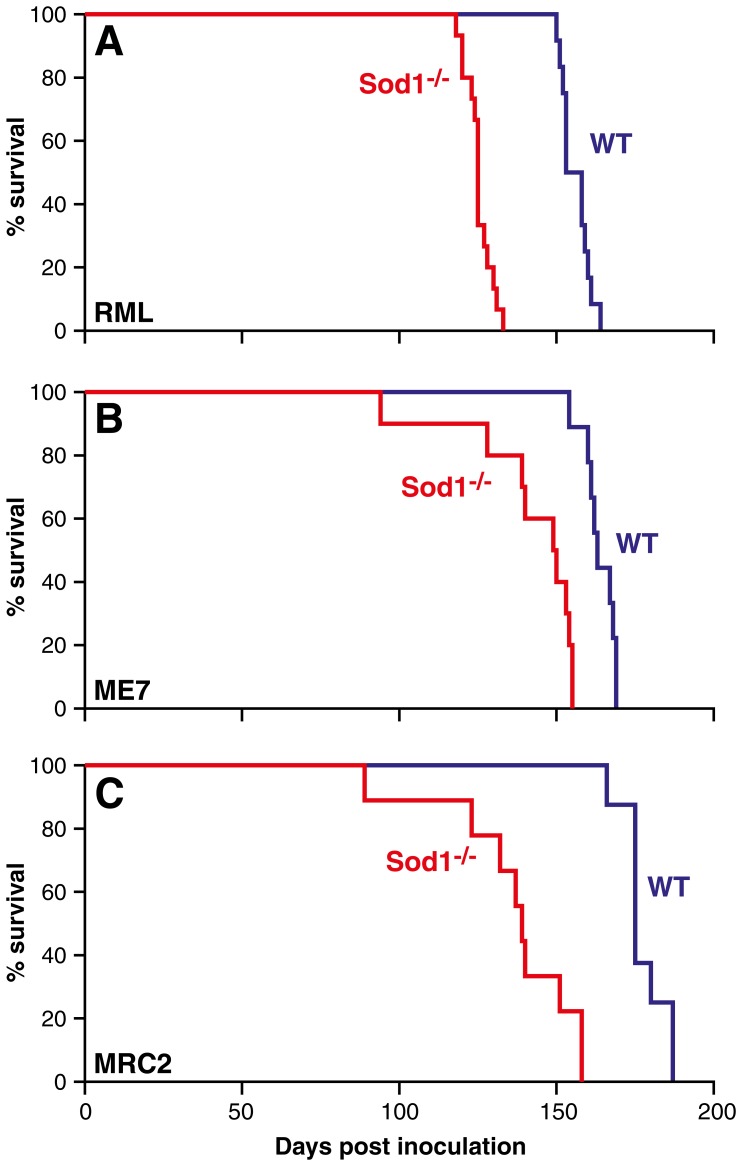
Kaplan-Meier survival curves. Data are shown as % of animals (female) surviving (y-axis) plotted against the number of days post inoculation (x-axis). (A) Transmission of Chandler/RML prion strain to *Sod1^−/−^* and *Sod1^+/+^* (WT) litter mate controls (B) Transmission of ME7 prion strain to *Sod1^−/−^* and *Sod1^+/+^* (WT). (C) Transmission of MRC2 mouse adapted BSE prion strain to *Sod1^−/−^* and *Sod1^+/+^* (WT). A reduction in mean incubation time of 20%, 13%, and 24% was seen in A-C respectively. This reduction in survival was statistically significant for each transmission (P<0.001, Kaplan-Meier log-rank survival test).

It was previously reported that overexpression of human SOD1 in a transgenic mouse model increased prion disease incubation time with the RML prion strain, however, this was not replicated with the 301 V (BSE passaged in *Prnp^b^* allele mice, B6.I) strain [21]. These transmissions were carried out in small groups (n = 4 or 5), on a mixed genetic background and in the presence of a foreign protein, therefore, to clarify the role of Sod1 we transmitted three different prion strains to Sod1 deficient mice. We used *Sod1* knockout mice B6;129S7-Sod1^tm1Leb^/J backcrossed to C57BL/6J (*Sod1^−/−^*) and their litter mates (*Sod1^+/+^*) as wild type controls [27]. Although mutations in human SOD1 are associated with familial amyotrophic lateral sclerosis (fALS), *Sod1* deficient mice are neurologically normal and do not develop spontaneous neurodegenerative disease. The only phenotype associated with these mice is female sterility in homozygote knockouts [27]. We inoculated mice intracerebrally with two different mouse-adapted scrapie prion strains, Chandler/RML and ME7, and a mouse adapted BSE prion strain, MRC2 (BSE passaged in *Prnp^a^* mice) [28], [34]. In *Sod1^−/−^* mice a significant decrease in incubation time was seen for all three prion strains as compared to *Sod1^+/+^* controls (Figure 2 and Table S2). For Chandler/RML prions the mean incubation time (days ± sem) was reduced by 20% from 156±1 in *Sod1^+/+^* to 125±1 in *Sod1^−/−^* mice (P<0.0001, Kaplan-Meier log rank survival) and for ME7 the mean incubation time was reduced by 13% from 164±2 in *Sod1^+/+^* to 142±5 in *Sod1^−/−^* mice (P<0.0001, Kaplan-Meier log rank survival). For MRC2 the mean incubation time was reduced by 24% from 178±2 in *Sod1^+/+^* to 136±7 in *Sod1^−/−^* mice (P<0.0001, Kaplan-Meier log rank survival). These data were obtained using female mice, however, similar results were also recorded for male mice although the mean differences were reduced to 16, 5 and 13% for Chandler/RML, ME7 and MRC2 prion strains respectively (Table S2).

**Figure 3:**
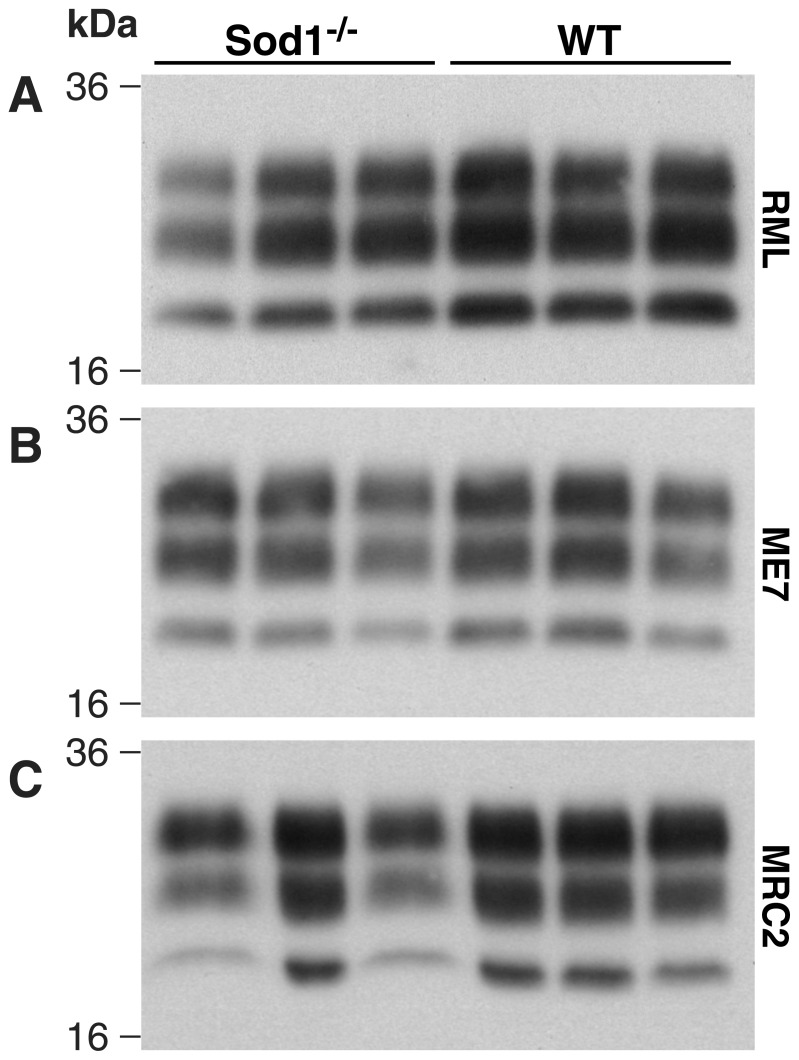
Western blots of PrP^Sc^ from infected mouse brains. 10% w/v brain homogenates (n = 3 per group) were digested with proteinase K and immunoblotted with anti-PrP monoclonal antibody ICSM35 (D-Gen Ltd, UK). (A) Transmission of Chandler/RML prions to *Sod1^−/−^* and *Sod1^+/+^* (WT) litter mate controls. (B) Transmission of ME7 prion strain to *Sod1^−/−^* and *Sod1^+/+^* (WT) litter mate controls. (C) Transmission of MRC2 mouse adapted BSE prion strain to *Sod1^−/−^* and *Sod1^+/+^* (WT) litter mate controls. No differences were seen between the two groups regardless of prion strain.

**Figure 4:**
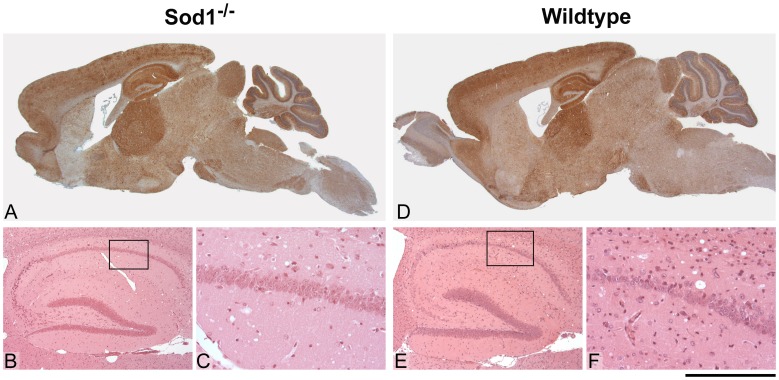
RML histology. Histological features of Chandler/RML prion transmission to *Sod1^−/−^* (A–C) and wild type control (D–F) mice. Panels A and D show distribution of disease-associated PrP by immunohistochemistry using anti-PrP monoclonal antibody ICSM35. Panels B, C, E and F show detail from the hippocampus and are stained with haematoxylin and eosin (H&E) to visualise spongiform change and neuronal loss. There is almost no neuronal loss in the *Sod1^−/−^* mice but mild neuronal loss is seen in the wild type animals. Overall, the pattern of spongiosis, gliosis and PrP distribution are similar between the two groups, however, the distribution of disease-associated PrP is patchier in the knockouts especially in the cortex. Scale bar corresponds to 3 mm (A, D), 660 µm (B, E) or 160 µm (C, F).

Prion strains can be differentiated by biochemical differences in PrP^Sc^ or by patterns of neuropathology in recipient animals. Western blotting of brains from infected animals confirmed that there is no change to the PrP^Sc^ type caused by replication in the absence of Sod1 (Figure 3). Pathological evaluation was carried out on brain sections from *Sod1^−/−^* and *Sod1^+/+^* mice for each transmission with particular attention to PrP^Sc^ deposition, spongiosis, gliosis and neuronal loss (Figure 4, Figures S1 and S2). For Chandler/RML transmission, the pattern of gliosis and PrP^Sc^ staining is similar, however, PrP^Sc^ staining is patchier for the *Sod1^−/−^* particularly in the cortex. No hippocampal neuronal loss is seen in *Sod1^−/−^* mice while mild neuronal loss is seen in wild type animals. No differences are seen between the two groups of mice following ME7 infection. For MRC2 transmission there is no difference in the pattern of gliosis and PrP^Sc^ deposition, however, there is a reduced intensity of staining especially in the cortex stripe in the *Sod1^−/−^* mice as compared to wild type. Neuronal loss in the hippocampus is very variable for the MRC2 transmissions, however, there is no clear difference between the two groups of mice.

**Figure 5:**
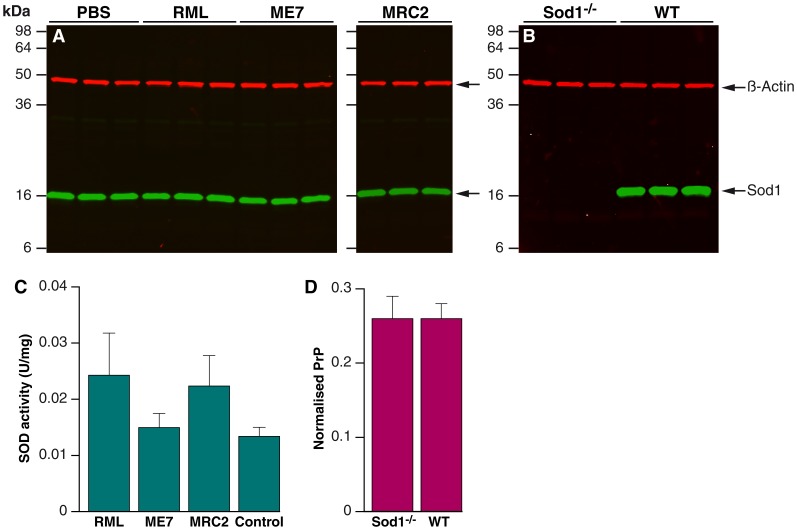
Quantification of Sod and PrP^C^ . 10% weight/volume brain homogenates immunoblotted with rabbit polyclonal anti-human SOD1 (Abcam) and detected with IRDye800CW (green) conjugated goat anti-rabbit IgG (Li-Cor). Anti-β-actin mouse monoclonal antibody (Sigma) was also included as a loading control and was detected using IRDye680 (red) conjugated goat anti-mouse IgG (Li-Cor). Fluorescence was visualised using an Odyssey infra-red imager (Li-Cor). (A) Brains from *Sod1^+/+^* wild type mice inoculated with PBS compared with end stage Sod1^+/+^ wild type mice inoculated with RML, ME7 and MRC2 prion strains. No differences were seen between the groups. (B) Uninfected mice. (C) Total SOD enzymatic activity in 10% (w/v) *Sod1^+/+^* (WT) brains. Brains from terminally sick mice infected with RML, ME7 and MRC2 were compared with uninfected mice. Samples were run in triplicate with n = 6 for each group. Data are shown normalised by total protein content (µg/ml) as determined by a Bradford protein assay (mean ± standard deviation). No significant difference was seen between the groups. (D) PrP^c^ levels in *Sod1^−/−^* and *Sod1^+/+^* (WT) litter mate control mice by ELISA. PrP^c^ levels (µg/ml) were determined in triplicate using 10% (weight/volume) brain homogenate for *Sod1^−/−^* (n = 3) and *Sod1^+/+^* (n = 3) in a PrP specific ELISA [32]. Data are shown normalised by total protein content (µg/ml and x 1000) as determined by a BCA assay (mean ± standard deviation). No significant difference was seen between the two groups.

We have shown that variation at the *Sod1* locus is associated with prion disease incubation time and that this is not explained by differences in mRNA expression levels. We therefore looked at Sod1 protein levels by western blot in *Sod1^+/+^* control mice infected with Chandler/RML, ME7 and MRC2 prions and compared these with age matched controls (PBS inoculated mice). No differences were seen between the four groups (Figure 5A). We also confirmed that the *Sod1^−/−^* mice used in these experiments did not express any detectable Sod1 protein (Figure 5B). Although the amount of Sod1 appears the same in uninfected and infected mice it is possible that Sod enzymes are more active in the environment created by prion infection. We therefore measured total Sod enzymatic activity in *Sod1^+/+^* mice infected with each of three prion strains and compared these to uninfected animals. No significant differences were seen between the groups (Figure 5C).

In experimental mouse models the level of PrP^c^ expression has a profound effect on incubation time therefore it is possible that the reduction in incubation time observed in *Sod1^−/−^* mice could be the result of an upregulation of the endogenous levels of PrP^c^ [2], [3]. We tested this by measuring total PrP^c^ levels in a PrP specific ELISA in Sod1^−/−^ and *Sod1^+/+^* mice [32]. No significant difference was observed (Figure 5D).

## Discussion

Quantitative trait loci mapping in mice and GWAS in human have confirmed that multiple genes in addition to *Prnp* influence prion disease incubation time and susceptibility [13], [15], [17], [18], [20]. These studies have successfully identified candidate loci but no individual genes have yet been confirmed as functionally significant. Alternative strategies have included targeting genes from pathways implicated in prion disease and testing them directly in mouse models. This identified *App*, *Il1-r1* and *Sod1* as interesting candidates, however, it did not address whether or not they contributed to the natural variation [21]. In this study we carried out an association study for each of these genes in HS of mice. The mapping resolution available in the HS cross at generation 37 is approximately 1–2 cM which is too big to unambiguously ascribe an association to one gene although mapping to SDPs may help to narrow down the region [25].

*App* is a very promising candidate gene particularly given its involvement in Alzheimer’s disease (AD), a neurodegenerative protein misfolding disease. There is also increasing evidence for an interaction with PrP where PrP has been implicated in post-translational processing of APP through the inhibition of BACE1 and in some mouse models PrP may bind amyloid-β and mediate aspects of its neurotoxicity [35]–[38]. Despite its attractions as a candidate we did not find any evidence of an association with prion disease incubation time in this cross although it is possible that other SDPs not tested by us may show some association. *App* may still have a role in prion disease although it does not appear to contribute to the natural variation observed in the parental lines of this cross.

Il1-r1 is a receptor for the pro-inflammatory cytokine, Il-1, produced by glia and implicated in the disease process as a result of extensive gliosis. *Il1-r1* knockout mice have been previously studied confirming that deletion of *Il1-r1* is protective in models of mouse scrapie [21], [39]. In this study we identify a non-synonymous SNP (Ala71Val) on a SDP that is linked to prion disease incubation time. It is not clear whether this SNP is the functional variant or whether it is carried within the same haplotype block.

Sod1 is an ubiquitously expressed cytoplasmic superoxide dismutase that protects cells from oxidative damage by removing superoxide through converting it to hydrogen peroxide. It has been associated with other neurodegenerative diseases notably amyotrophic lateral sclerosis where mutations in human SOD1 account for ∼20% of familial ALS cases probably via a toxic gain of function [40]. Sod1 has also been implicated in Alzheimer’s disease whereby *Sod1* knockout accelerated amyloid-β oligomerisation and memory loss in an AD mouse model and conversely overexpression of Sod1 rescues the cerebral endothelial dysfunction in another AD model [41], [42]. In this study we have shown a highly significant association with the *Sod1* locus and prion disease incubation time thereby confirming its effect on the natural variation within these inbred lines of mice. The functional SNPs remain unknown and we have not detected any evidence for differential mRNA or protein expression. We have also confirmed the role of Sod1 as a protective factor in prion disease as deletion of Sod1 significantly reduces incubation time in two different strains of mouse adapted scrapie (Chandler/RML and ME7) and a mouse passaged BSE prion strain, MRC2. These data broadly agree with previously published data that suggested a protective effect of overexpressing human SOD1 in mice challenged with RML but not 301 V prions [21]. This difference may be explained by the presence of the human protein or by differences in the mouse adapted BSE strains caused by passage in *Prnp^b^* rather than *Prnp^a^* mice. We cannot exclude the possibility that the targeted knockout of Sod1 in these mice may inadvertently affect the expression of adjacent loci which themselves influence prion disease incubation time and that this would explain the absence of detectable differences in Sod1 expression.

Oxidative stress has been implicated in neurodegenerative diseases and is caused by excess reactive oxygen species such as superoxide accumulating with the cell which may be the result of excess production by malfunctioning mitochondria and depletion of catalytic enzymes. In mouse models of prion disease damaged mitochondria have been described as well as evidence for increased superoxide generation and free radical damage [43], [44]. Markers of oxidative damage have also been described in CJD brains [45]. We did not detect a change in Sod1 protein expression or total Sod activity in response to prion infection with any of our prion strains, however, the presence of normal Sod1 activity is clearly protective as Sod1 deficient mice show a significantly reduced incubation time across all prion strains tested.

### Conclusion

In conclusion, we have shown that both *Il1-r1* and *Sod,* but not *App,* are associated with the natural variation in prion disease incubation time measured in the Northport HS mice. In addition, Sod1 deficient mice show highly significant reductions in incubation time of 20, 13 and 24% with Chandler/RML, ME7 and MRC2 prion strains, respectively. Although no differences in Sod1 expression or activity were detected our data highlight the importance of oxidative damage in prion disease and the protective role played by endogenous Sod1 protein.

## Supporting Information

Figure S1 **ME7 histology.** Histological features of ME7 prion transmission to *Sod1^−/−^* (A–C) and wild type control (D–F) mice. Panels A and D show PrP^Sc^ distribution by staining with anti-PrP monoclonal antibody ICSM35. Panels B, C, E and F show detail from the hippocampus and are stained with haematoxylin and eosin (H&E) to visualise spongiform change and neuronal loss. No differences are seen between the two groups. Scale bar corresponds to 3 mm (A, D), 660 µm (B, E) or 160 µm (C, F).
(TIF)

Figure S2 **MRC2 histology.** Histological features of MRC2 prion transmission to *Sod1^−/−^* (A–C) and wild type control (D–F) mice. Panels A and D show PrP^Sc^ distribution by staining with anti-PrP monoclonal antibody ICSM35. Panels B, C, E and F show detail from the hippocampus and are stained with haematoxylin and eosin (H&E) to visualise spongiform change and neuronal loss. *Sod1^−/−^* mice (D) show a reduction in PrP intensity relative to wild type mice especially in the cortex stripe. Scale bar corresponds to 3 mm (A, D), 660 µm (B, E) or 160 µm (C, F).
(TIF)
