# Supplementary figures and images for: Sod1 Deficiency Reduces Incubation Time in Mouse Models of Prion Disease
Source: PLoS One. 2013 Jan 22;8(1):e54454. doi: 10.1371/journal.pone.0054454 (PMC3551847; doi:10.1371/journal.pone.0054454)

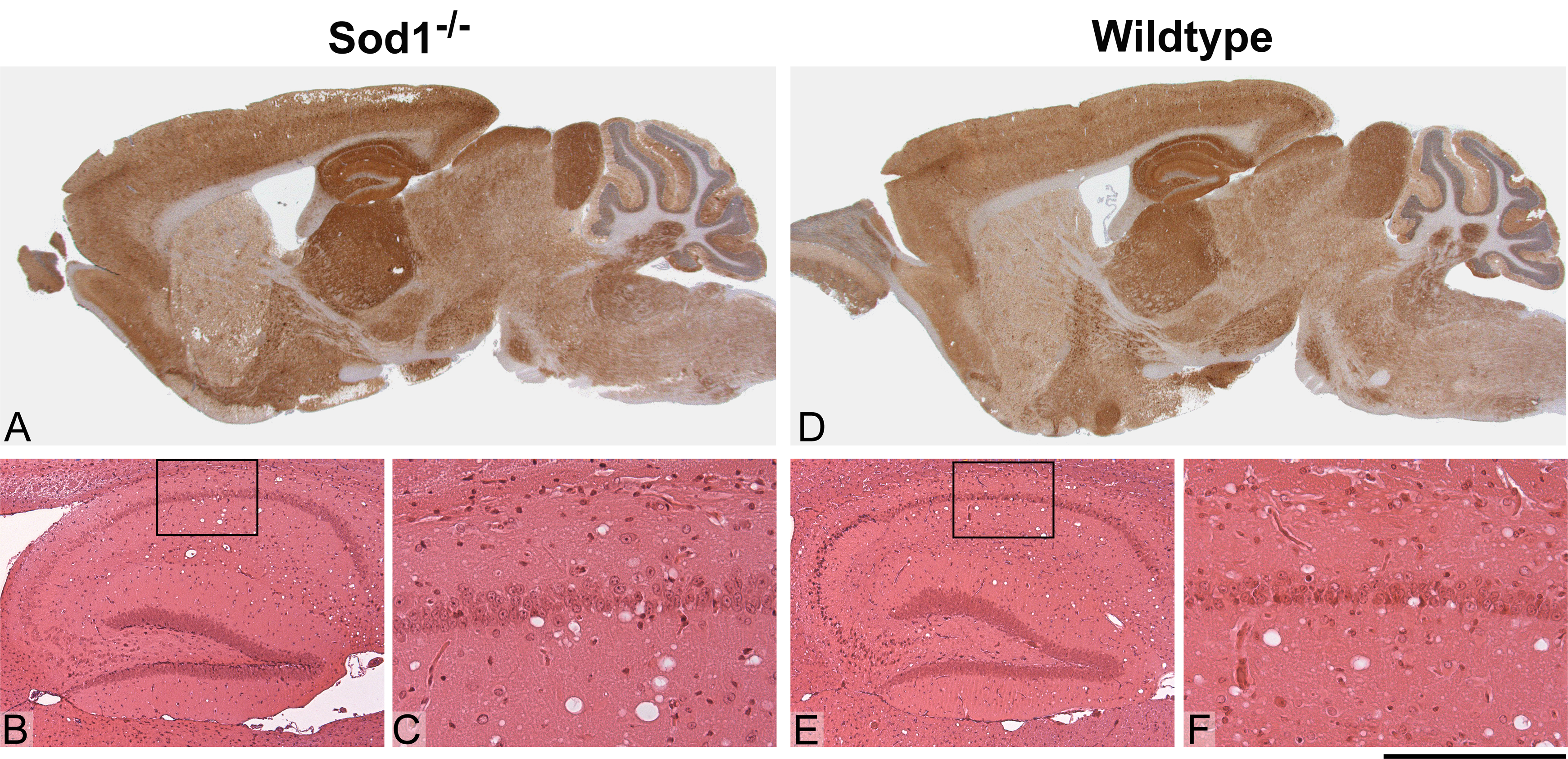

Supplement: Figure S1 — ME7 histology. Histological features of ME7 prion transmission to Sod1−/− (A–C) and wild type control (D–F) mice. Panels A and D show PrPSc distribution by staining with anti-PrP monoclonal antibody ICSM35. Panels B, C, E and F show detail from the hippocampus and are stained with haematoxylin and eosin (H&E) to visualise spongiform change and neuronal loss. No differences are seen between the two groups. Scale bar corresponds to 3 mm (A, D), 660 µm (B, E) or 160 µm (C, F). (TIF) [file pone.0054454.s001.tif]

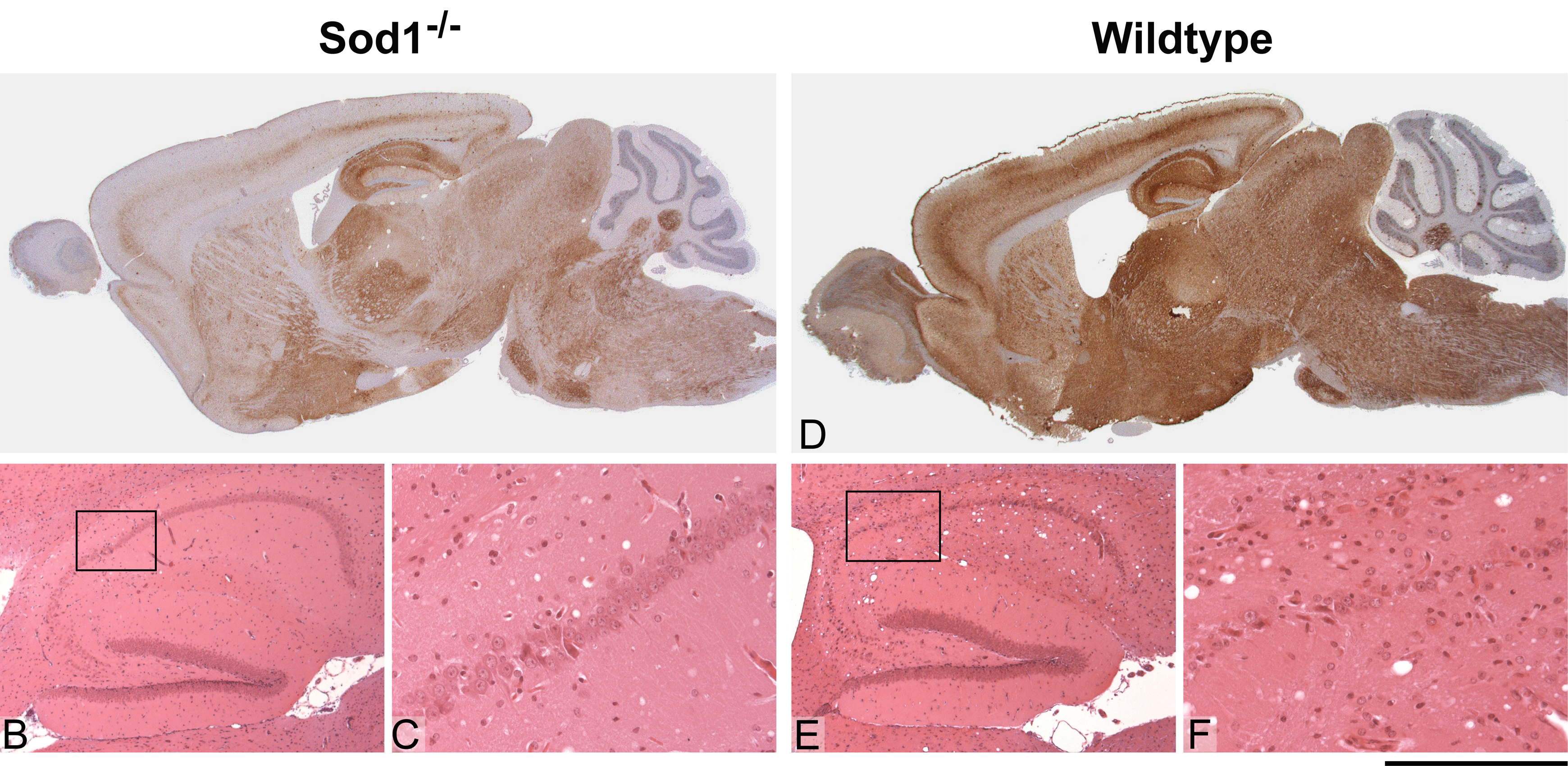

Supplement: Figure S2 — MRC2 histology. Histological features of MRC2 prion transmission to Sod1−/− (A–C) and wild type control (D–F) mice. Panels A and D show PrPSc distribution by staining with anti-PrP monoclonal antibody ICSM35. Panels B, C, E and F show detail from the hippocampus and are stained with haematoxylin and eosin (H&E) to visualise spongiform change and neuronal loss. Sod1−/− mice (D) show a reduction in PrP intensity relative to wild type mice especially in the cortex stripe. Scale bar corresponds to 3 mm (A, D), 660 µm (B, E) or 160 µm (C, F). (TIF) [file pone.0054454.s002.tif]
